# Association between dietary inflammatory index and musculoskeletal disorders in adults

**DOI:** 10.1038/s41598-023-46429-w

**Published:** 2023-11-20

**Authors:** Firoozeh Khamoushi, Davood Soleimani, Farid Najafi, Neshat Ahmadi, Neda Heidarzadeh-Esfahani, Bita Anvari, Ebrahim Shakiba, Yahya Pasdar

**Affiliations:** 1 Student Research Committee, Department of Nutritional Sciences, School of Nutritional Sciences and Food Technology, Kermanshah University of Medical Sciences, Kermanshah, Iran; 2 Nutritional Sciences Department, School of Nutrition Sciences and Food Technology, Kermanshah University of Medical Sciences, Kermanshah, Iran; 3 Research Center of Oils and Fats, Kermanshah University of Medical Sciences, Kermanshah, Iran; 4 Research Center for the Environmental Determinants of Health (RCEDH), Health Institute, Kermanshah University of Medical Sciences, Kermanshah, Iran; 5 Cardiovascular Research Center, Kermanshah University of Medical Sciences, Kermanshah, Iran; 6 Internal Medicine Department, Imam Khomeini Hospital, School of Medicine, Kermanshah University of Medical Sciences, Kermanshah, Iran; 7 Social Development and Health Promotion Research Center, Kermanshah University of Medical Sciences, Kermanshah, Iran

**Keywords:** Diseases, Rheumatology

## Abstract

This research investigated how the Dietary Inflammatory Index (DII) related to musculoskeletal issues in adults. It used a cross-sectional design with a sample of 3477 female and 3572 male participants aged 35 to 65 from the Ravansar Non-Communicable Diseases cohort study in western Iran. The DII is calculated from a Food Frequency Questionnaire (FFQ) to measure dietary intake. Musculoskeletal disorders including back pain, back pain/stiffness, joint pain, and joint pain/stiffness were evaluated by the RaNCD cohort study physician using a standard questionnaire. Logistic regression analysis examined the association between DII and musculoskeletal disorders. The findings demonstrated a positive association between higher DII scores and back pain/stiffness (OR 1.32, 95% CI 1.04–1.73, P = 0.047). Furthermore, DII displayed a significant association with a heightened odd to joint pain (OR 1.26, CI 1.10–1.46) when compared to those with lower DII scores (Q3 vs. Q1). After adjusting for cofounding factors, the Q3 DII quintile participants showed a 44% higher odd of experiencing joint pain/stiffness (OR 1.44, CI 1.01–2.05, P = 0.047). However, the study found no significant association between back pain and DII (P > 0.05). In conclusion, the research suggests that consuming a pro-inflammatory diet might be linked to developing musculoskeletal issues in adults.

## Introduction

Musculoskeletal disorders encompass a range of injuries or conditions that affect the body's musculoskeletal system, consisting of nerves, tendons, muscles, joints, ligaments, and cartilage^1^. In the year 2017, there existed an estimated 1.3 billion instances of musculoskeletal disorders on a worldwide scale. Consequently, 138.7 million disability-adjusted life years and 121.3 thousand fatalities were attributable to such conditions^2^.

Studies indicate that persons afflicted with musculoskeletal disorders commonly exhibit increased concentrations of cytokines, such as tumor necrosis factor (TNF), interleukin-1 (IL-1), and IL-6, along with typical inflammatory mediators like C-reactive protein (CRP)^3–6^. Identifying inflammation as a noteworthy factor in musculoskeletal pain has garnered considerable attention^7^. There exists a significant corpus of evidence reinforcing the notion that dietary factors play a pivotal role in the modulation of inflammation and potentially facilitate the onset of musculoskeletal pathologies^8–10^. Therefore, it has become imperative to confront this matter by effectively managing inflammation via dietary interventions^8–10^. Specific nutritional components, such as fruits, vegetables, whole grains, and spices, have demonstrated anti-inflammatory effects due to their rich antioxidant and polyphenol content^11^. On the other hand, high consumption of animal proteins, fats, sugar, dairy products, and refined carbohydrates has been linked to increased inflammation^12^. Some nutritional studies have indicated that anti-inflammatory foods like nuts, tea, fish, olive oil, and vegetables reduce inflammation and less severe musculoskeletal pain^13,14^. Therefore, an anti-inflammatory diet could be valuable in managing musculoskeletal conditions^7,15^.

The dietary inflammatory index (DII) can evaluate the overall nutritional pattern to gain more comprehensive insights into the diet-disease association^11,13^. Investigating the association between the DII and musculoskeletal pain is of great importance as it sheds light on how diet can impact the musculoskeletal health of adults. Previous studies have focused on exploring the association between the DII and factors such as handgrip strength and body composition (including fat mass, fat-free mass, and percent body fat)^16,17^. Although studies on this topic are limited, the results of a recent study performed on 212 elderly individuals showed that higher DII score was positively associated with musculoskeletal pain^7^. However, this current study is pioneering in the Western region of Iran, as it examines the link between DII and various types of pain, such as back pain, back pain/stiffness, joint pain, and joint pain/stiffness, among the adult population.

## Methods

### Study population

The present study was conducted using a cross-sectional research design and drew upon data from the ongoing non-communicable diseases cohort study (RaNCD) in Kermanshah province, in Western Iran. The study was specifically focused on individuals of Kurdish descent and has been ongoing since 2014. A total of 10,047 adults, comprising both males and females aged between 35 and 65 years, were recruited to participate in the study. Notably, the RaNCD project is a constituent of the more extensive Prospective Epidemiological Research Studies in Iran (PERSIAN) study, which has been granted ethical approval by Iran's Ministry of Health and Medical Education. Interested readers may consult earlier publications to comprehensively understand the PERSIAN and RaNCD cohort studies^18,19^. All the participants included in the current investigation were drawn from the RaNCD study's baseline phase, constituting a sample size of 10,047 individuals.

**Figure 1:**
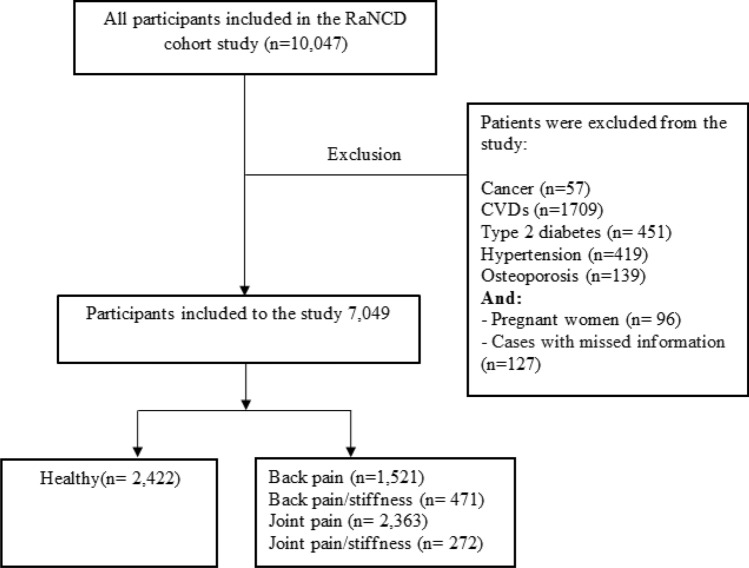
Flow diagram of the study participants.

After applying exclusion criteria, such as cardiovascular diseases, cancer, type 2 diabetes, hypertension, osteoporosis history, pregnancy, and incomplete information, the final sample size was reduced to 7049 participants (refer to Fig. 1). The data collection process involved face-to-face interactions at the RaNCD cohort site, where questionnaires, measurements, and tests were conducted and evaluated.

### Assessment of other variable

Data concerning various demographic factors and lifestyle aspects, such as age, gender, socio-economic status, place of residence, smoking habits, and alcohol consumption, were collected using digital questionnaires administered by trained interviewers. To capture information on chronic diseases, medications, and the use of NSAIDs (aspirin, ibuprofen, naproxen, celecoxib, ketorolac, diclofenac, indomethacin, and piroxicam), a medical history questionnaire was employed. Physical activity levels were evaluated through the PERSIAN cohort questionnaire, and participants' responses were measured in terms of the metabolic equivalent of task per hour per day (MET/h per day), following a methodology from a separate study^19^. The collected MET values were categorized into three tertiles: Light (< 3), Moderate (3–6), and High (≥ 6)^20,21^.

### Dietary inflammatory index (DII)

The DII score was computed for participants at the RaNCD study site through the completion of a 118-item Food Frequency Questionnaire (FFQ), which is known for its validity and reliability^22^. This questionnaire was based on the method developed by Shivappa et al.^23^ and focused on 45 food items that have been shown to influence inflammation, either positively or negatively. For this particular study, our focus was on 31 distinct food items, including onion, garlic, coffee, tea, energy, protein, carbohydrates, fiber, and various essential vitamins (A, C, D, E, B1, B2, B3, B6, B12, and folate), as well as beta-carotene, total fat, saturated fatty acids (SFAs), monounsaturated fatty acids (MUFAs), polyunsaturated fatty acids (PUFAs), omega-3 and omega-6 fatty acids, cholesterol, magnesium, iron, selenium, and zinc. To determine the DII score, we compared the intake of these selected food items to global mean and standard deviation data obtained from 11 worldwide studies^23^. The cumulative sum of these specific food items was then used to calculate the DII score. A positive DII score indicated a pro-inflammatory diet, while a negative score suggested an anti-inflammatory diet^23,24^. For analysis, we further categorized the DII scores into quartiles, with the first and fourth quartiles representing the lowest and highest DII scores, respectively.

### Outcomes

The RaNCD cohort study evaluated musculoskeletal conditions, encompassing a range of disorders like back pain. Back pain was defined as the experience of continuous back pain lasting over a week and significantly interfering with daily activities. Additionally, the study included back pain/stiffness, which referred to back pain accompanied by morning stiffness lasting an hour or more, as well as joint pain, indicating a history of experiencing pain in the joints. Moreover, standard pain/stiffness was also assessed, which reflected a history of joint pain and morning stiffness lasting an hour or more. To evaluate these conditions, physicians assessed the participants and asked specific questions to which the participants responded.

The questions asked were as follows:

1. Have you ever experienced low back pain lasting over a week, significantly disrupting your daily activities? (Yes/No).
2. Have you experienced back stiffness for more than an hour in the morning? (Yes/No).
3. Have you had arthralgia? (Yes/No).
4. Have you experienced joint stiffness for more than an hour in the morning? (Yes/No).

It's worth noting that the study did not consider pain associated with fractures, malignancies, and infections as part of its definition of back pain.

### Statistical analysis

For data analysis, the researchers utilized STATA version 14.2 software (Stata Corp, College Station, TX, USA). They employed the ANOVA test to assess the variations in continuous variables across quartiles of the DII. For categorical variables, the chi-square test was used.The researchers conducted logistic regression to determine the odds ratios (ORs) and 95% confidence intervals (CIs) for the impact of back pain, back pain/stiffness, joint pain, and joint pain/stiffness. The analysis involved three models:

1. Model 1, the crude model, examined the associations without any adjustments.
2. Model 2 included adjustments for age and gender.
3. Model 3 further incorporated adjustments for age, gender, BMI, physical activity, energy intake, and socio-economic status (SES).

Statistical significance was indicated by a p-value below 0.05, and 95% confidence intervals (CIs) were calculated.

### Ethics approval and consent to participate

The Ethics Committee of Kermanshah University of Medical Sciences approved the study (KUMS.REC.1394.318). All methods were carried out in accordance with relevant guidelines and regulations. All the participants were provided oral and written informed consent.

## Results

The study examined participant characteristics based on DII quartiles, as shown in Table 1. Various variables, such as age, gender, place of residence, job, socio-economic status, alcohol consumption, BMI, WHR, energy intake, protein, fat, fiber, zinc, calcium, Fe, B6, B12, and physical activity, exhibited significant differences (p < 0.001). The average DII quartiles ranged from − 4.03 ± 0.40 (Q1: indicating the most anti-inflammatory diet) to − 0.10 ± 1.06 (Q4: suggesting the most pro-inflammatory diet). Urban residents were predominantly found in Q4, demonstrating a significant difference in the DII quartiles (p < 0.001). Furthermore, 39.84% of participants who consumed alcohol were in the highest DII quartile (the most pro-inflammatory diet) (p < 0.001). Participants with a higher socio-economic status also showed a notably higher DII (p < 0.001). The fourth quartile displayed a significantly higher average energy intake (3398 ± 971.78), protein (14.45 ± 2.19), fat (27.20 ± 5.59), fiber (35 ± 11.12), zinc (14.12 ± 4.93), calcium (1651.93 ± 612.86), Fe (24.38 ± 8.60), B6 (17.35 ± 11.85), and B12 (11.61 ± 8.09), compared to the first quartile (p < 0.001). Moreover, individuals with a higher BMI were more prevalent in Q4 (the most pro-inflammatory diet). The prevalence of back pain, back pain/stiffness, joint pain, and joint pain/stiffness varied across DII quartiles, with the majority in the third and fourth quartiles. However, only the percentage of back pain/stiffness showed statistical significance (p < 0.001) (Table 1).

**Table 1:** Characteristics of participants according to quartiles of the dietary inflammatory index score.

Variable	Dietary inflammatory index (DII)				P value trend*
	Quartile 1: most anti-inflammatory	Quartile 2	Quartile 3	Quartile 4: most pro-inflammatory	
Frequency, n	1711	1771	1782	1785	–
DII, mean ± SD	− 4.03 ± 0.40	− 3.13 ± 0.25	− 2.10 ± 0.38	− 0.10 ± 1.06	–
Age, mean ± SD	46.64 ± 7.96	45.81 ± 7.73	44.93 ± 7.45	44.62 ± 7.29	< 0.001
Gender, n (%)					
Male	730 (20.44)	834 (23.35)	929 (26.01)	1079 (30.21)	< 0.001
Female	981 (28.21)	937 (26.95)	853 (24.53)	706 (20.30)	
Place of residence, n (%)					
Urban	635 (15.18)	913 (21.83)	1212 (28.98)	1422 (34.00)	< 0.001
Rural	1076 (37.53)	858 (29.93)	570 (19.88)	363 (12.66)	
Job, n (%)					
Unemployed	24 (26.37)	28 (30.77)	18 (19.78)	21 (23.08)	< 0.001
Employed	747 (20.32)	861 (23.42)	976 (26.54)	1093 (29.73)	
Retired	25 (20.49)	32 (26.23)	36 (29.51)	29 (23.77)	
Housewife	914 (28.94)	850 (26.92)	752 (23.81)	642 (20.33)	
Smoking, n (%)					
Never	1380 (24.58)	1428 (25.43)	1420 (25.29)	1387 (24.70)	0.280
Current smoker	203 (22.83)	227 (25.53)	223 (25.08)	236 (26.55)	
Former smoker	125 (24.32)	109 (21.21)	127 (24.71)	153 (29.77)	
Use of alcohol					
Yes	57 (15.45)	74 (20.05)	91 (24.66)	147 (39.84)	< 0.001
No	1654 (24.76)	1697 (25.40)	1691 (25.31)	1638 (24.52)	
Socio-economic status, n (%)					
Low	768 (34.77)	594 (26.89)	416 (18.83)	431 (19.51)	< 0.001
Moderate	498 (21.20)	593 (25.24)	628 (26.73)	630 (26.82)	
High	444 (17.84)	584 (23.46)	738 (29.65)	723 (29.05)	
Physical activity (Met-h/day), n (%)					
Light	465 (23.07)	538 (26.69)	533 (26.44)	480 (23.81)	0.049
Moderate	856 (25.66)	815 (24.43)	861 (25.81)	804 (24.10)	
High	390 (22.98)	418 (24.63)	388 (22.86)	501 (29.52)	
BMI (kg/m^2^), mean ± SD	26.56 ± 4.56	26.98 ± 4.52	27.21 ± 4.60	27.49 ± 4.50	< 0.001
WHR, mean ± SD	0.93 ± 0.06	0.93 ± 0.06	0.94 ± 0.06	0.94 ± 0.06	< 0.001
WC (cm)	96.10 ± 10.46	96.36 ± 10.31	96.57 ± 10.43	95.96 ± 10.18	0.588
VFA (cm^2^)	113.50 ± 49.65	116.71 ± 50.24	117.80 ± 51.27	116.22 ± 50.91	0.128
Energy intake (Kcal/day)	2184.75 ± 688.32	2412.50 ± 693.53	2776.32 ± 793.90	3398 ± 971.78	< 0.001
Protein (%Kcal/day)	13.10 ± 1.90	13.48 ± 2.03	13.76 ± 2.09	14.45 ± 2.19	< 0.001
Carbohydrates (%Kcal/day)	61.13 ± 6.65	61.53 ± 5.98	61.31 ± 5.92	61.05 ± 5.95	0.257
Fat (%Kcal/day)	26.89 ± 6.45	26.69 ± 5.89	27.04 ± 5.70	27.20 ± 5.59	0.012
Fiber (g/day)	16.06 ± 5.59	20.09 ± 6.01	25.48 ± 7.03	35.44 ± 11.12	< 0.001
Zinc (mg/day)	7.82 ± 3.07	8.90 ± 2.90	10.71 ± 3.53	14.12 ± 4.93	< 0.001
Calcium (mg/day)	1101.46 ± 455.27	1176.21 ± 477.23	1337.66 ± 509.02	1651.93 ± 612.86	< 0.001
Fe (mg/day)	14.74 ± 5.29	16.38 ± 5.94	18.85 ± 6.50	24.38 ± 8.60	< 0.001
B6 (mg/day)	6.79 ± 5.47	9.07 ± 7.17	11.90 ± 8.59	17.35 ± 11.85	< 0.001
B12 (µg/day)	4.82 ± 3.89	5.81 ± 3.96	7.74 ± 4.96	11.61 ± 8.09	< 0.001
NSAID, n (%)	30 (28.04)	22 (20.56)	26 (24.30)	29 (27.10)	0.314
Back pain, n (%)	378 (24.85)	378 (24.85)	389 (25.58)	376 (24.72)	0.551
Back pain/stiffness, n (%)	97 (20.59)	93 (19.75)	150 (31.85)	131 (27.81)	0.002
Joint pain, n (%)	541 (22.89)	592 (25.05)	635 (26.87)	595 (25.18)	0.154
Joint pain/stiffness, n (%)	60 (22.06)	63 (23.16)	83 (30.51)	66 (24.26)	0.423

*BMI* body mass index, *WHR* waist/hip ratio, *WC* waist circumference, *VFA* visceral fat area, *NSAID* nonsteroidal anti-inflammatory drug. *Analysis of variance (ANOVA) and Chi-squared test, P < 0.05.

### DII and back pain, back pain/stiffness, joint pain, and joint pain/stiffness

The study's results indicate that odds ratios are associated with back pain/stiffness and joint pain/stiffness concerning the DII quartiles. For back pain/stiffness, the odd was 1.32 times higher in the fourth DII quartile compared to the first quartile (OR 1.32, CI 1.04–1.73), and this association remained significant in models 2 and 3. Interestingly, no significant association was found between back pain and DII (p > 0.05).

Regarding joint pain, individuals in the third DII quartile had a 1.20 times higher odds ratio than the first. This association persisted after controlling for confounding variables in models 2 and 3 (OR 1.35, CI 1.17, 1.56; OR 1.26, CI 1.10–1.46, respectively). Additionally, the most pro-inflammatory diet in the fourth quartile showed a significant association with joint pain in model 2 (OR 1.30, CI 1.12–1.50).

After adjusting for confounding variables, the study found a direct association between DII and joint pain/stiffness. In the third DII quartile, the odds ratio of joint pain/stiffness was 1.44 times higher than in the first quartile (OR 1.44, CI 1.01–2.05) (Table 2).

**Table 2:** Crude and adjusted odds ratios (95% CIs) for musculoskeletal disorders across the quartiles of dietary inflammatory index (DII).

Outcome	Dietary inflammatory index (DII)	Model 1		Model 2		Model 3	
		OR (95% CI)	p value	OR (95% CI)	p value	OR (95% CI)	p value
Back pain	Quartile 1	1	–	1	–	1	–
	Quartile 2	0.96 (0.83, 1.12)	0.586	0.97 (0.83, 1.14)	0.746	0.96 (0.81, 1.13)	0.622
	Quartile 3	0.98 (0.84, 1.15)	0.844	1.02 (0.87, 1.20)	0.790	0.99 (0.83, 1.17)	0.872
	Quartile 4	0.94 (0.80, 1.12)	0.455	1.01 (0.84, 1.16)	0.920	1.01 (0.75, 1.20)	0.297
Back pain/stiffness	Quartile 1	1	–	1	–	1	–
	Quartile 2	0.92 (0.69, 1.23)	0.584	0.95 (0.71, 1.28)	0.744	0.92 (0.68, 1.23)	0.572
	Quartile 3	1.53 (1.17, 1.99)	0.002	1.65 (1.26, 2.15)	< 0.001	1.58 (1.19, 2.10)	0.001
	Quartile 4	1.32 (1.04, 1.73)	0.047	1.51 (1.14, 1.99)	0.003	1.52 (1.11, 2.07)	0.008
Joint pain	Quartile 1	1	–	1	–	1	–
	Quartile 2	1.10 (0.94, 1.25)	0.260	1.15 (0.99, 1.33)	0.057	1.11 (0.96, 1.28)	0.173
	Quartile 3	1.20 (1.04, 1.37)	0.013	1.35 (1.17, 1.56)	< 0.001	1.26 (1.10, 1.46)	0.003
	Quartile 4	1.10 (0.94, 1.24)	0.285	1.30 (1.12, 1.50)	< 0.001	1.13 (0.95, 1.33)	0.161
Joint pain/stiffness	Quartile 1	1	–	1	–	1	–
	Quartile 2	1.01 (0.70, 1.45)	0.938	1.10 (0.75, 1.54)	0.679	1.03 (0.72, 1.49)	0.861
	Quartile 3	1.34 (0.94, 1.88)	0.088	1.54 (1.10, 2.14)	0.013	1.44 (1.01, 2.05)	0.047
	Quartile 4	1.06 (0.74, 1.50)	0.076	1.31 (0.91, 1.88)	0.141	1.22 (0.88, 1.84)	0.368

*Model 1* Crude, *Model 2* Adjusted for age and gender, *Model 3* Adjusted for age, gender, BMI, physical activity, energy intake and SES.

## Discussion

The main outcome of this research is the presence of a direct relationship between the degree of dietary inflammation and back pain/stiffness and joint pain/stiffness, independent of potential confounders such as age, gender, BMI, energy intake, and physical activity levels. However, it is essential to mention that this relationship did not follow a dose–response pattern.

The relevant studies on this topic are scarce, and those which do exist are often on unhealthy population. The study conducted by Correa-Rodríguez et al. focused on menopausal women with fibromyalgia syndrome. It highlighted that the DII showed a significant association with increased pressure pain thresholds in some sites such as knee^24^. Another investigation by Toopchizadeh et al. involved 220 knee osteoarthritis patients and revealed a positive link between higher DII scores and increased pain levels based on the visual analog scale. They also have demonstrated a detrimental association between the DII scores and physical function and emotional well-being, independent of potential confounding variables such as age, gender, body mass index, and physical activity levels^25^. Moreover, Strath and colleagues have reported that the DII score was linked to the severity of movement-evoked pain in women afflicted with chronic back pain, although no such association was observed in men^26^. Similarly, the results of a recent study performed on 212 elderly individuals showed that grater DII score was positively associated with intense musculoskeletal pain^7^. Also, evidence shows that chronic low back pain may be related to lumbar vertebral bone mineral density (BMD) among community-dwelling middle-aged adults^27^. Although Cervo and colleagues observed a significant association between DII scores and lumbar spine BMD (B 0.013; 95% CI − 0.024 to − 0.002) in community-dwelling Australian older men, they didn’t find this association in women^28^. Sakai and colleagues revealed that low back pain had a negative correlation with skeletal muscle mass rather than with BMD^29^. Furthermore, Eguchi, et al. and Kim, et al. reported that chronic low back pain was positively associated with sarcopenia^30,31^. Therefore, musculoskeletal disorders are greatly influenced by sarcopenia and muscle mass. Chen and colleagues found that adherence to diets with high DII scores is significantly associated with a lower muscle mass and strength and higher risk for sarcopenia in older US adults^32^. Esmaeily and colleagues showed that higher DII scores were significantly associated with higher odds of sarcopenia and lower handgrip strength in community-dwelling older subjects^33^. Taken together, a recent meta-analysis on 24 studies involving 56,536 participants revealed that high DII scores increase the odds of low skeletal muscle mass, low skeletal muscle strength, and sarcopenia^34^.

Epidemiological investigations consistently demonstrate that dietary patterns characterized by a low DII, denoting an anti-inflammatory dietary regimen, are linked with a diminished incidence of non-communicable chronic ailments featuring inflammatory etiologies. Prior research has additionally established a correlation between DII scores and the probability of developing Rheumatoid arthritis^35^ and the quantity of tender and swollen joints present in individuals with this ailment^36^. Similarly, Sköldstam et al. found that adhering to a Mediterranean diet, known for its anti-inflammatory properties, was negatively associated with pain levels in patients with rheumatoid arthritis^37^. Specific dietary components like omega-3 polyunsaturated fatty acids^38^ and polyphenols have also been shown promising effects in animal models' reduction of intervertebral disc degeneration^39^. Disc degeneration is the main cause of morning back stiffness and pain among healthy subjects^40^.

A possible mechanism of inflammation reduction by anti-inflammatory diet could be through the reduction of prostaglandins, interacting with neuromodulator pathways (gamma-aminobutyric acid receptor signaling), inhibiting inflammatory signaling, focusing on l-arginine/nitric oxide signaling, and decreasing enzyme activity such as cyclooxygenase 2^7^. Chronic systemic inflammation can interrupt muscle homeostasis through suppressing insulin-like growth factor 1 (IGF-1) mediated by activation of the ubiquitin–proteasome system^41^. Also, elevated levels of inflammatory mediators such as interleukin 1-1β, tumor necrosis factor α, Interferon-γ in blood flow can lead to skeletal-muscle wasting through impairing the regenerative function of muscle stem cells and inducing accumulation of extracellular matrix and subsequently muscle fibrosis^42,43^. Hence, including anti-inflammatory factors in one's diet may be linked to a reduced risk of experiencing back pain and stiffness by lowering inflammation, minimizing intervertebral disc degeneration, altering pressure pain thresholds, and preventing skeletal-muscle weakness and wasting.

The key strength of this study lies in its utilization of data from a large population of a race and region. However, several limitations need to be acknowledged. Firstly, this research cannot reveal the causal relationship because of the nature of the cross-sectional study design, and it necessitates longitudinal studies to validate and establish the results conclusively. Secondly, the recall and self-reporting of outcomes in this study are vulnerable to information bias. Inaddition, people suffering from moderate to severe pain usually follow healthy dietary recommendations to reduce the symptoms of the disease. This phenomenon can affect and weaken the relationship between the dietary inflammatory index and pain. Thirdly, the absence of measurements for the origination and area of pain as well as the degree and intensity of pain hampers the interpretation of the findings. Furthermore, it is essential to note that the findings of this study cannot be readily applied to other populations due to specific factors. Notably, the participants in this study were highly active individuals engaged in physically demanding occupations such as farming and livestock farming, setting them apart from other groups. As a result, conducting further investigations while addressing the current study's limitations is highly recommended.

## Conclusion

The findings of this study lead to the deduction that there exists a significant association between the inflammatory characteristics of the diet and higher risk of having back pain/stiffness, as well as arthralgia among the subjects under examination. Consequently, it is advisable to commence dietary interventions at an early stage for the benefit of grownups who experience musculoskeletal problems.

## Data Availability

All data generated and analyzed during this study are included in the manuscript.

## Abbreviations

DII: Dietary inflammatory index
RaNCD: Ravansar non-communicable diseases cohort study
FFQ: Food frequency questionnaire
TNF: Tumor necrosis factor
IL-1: Interleukin-1
IL-6: Interleukin-6
CRP: C-reactive protein
